# Transgenic Mice for the Translational Study of Neuropathic Pain and Dystonia

**DOI:** 10.3390/ijms23158580

**Published:** 2022-08-02

**Authors:** Damiana Scuteri, Kengo Hamamura, Chizuko Watanabe, Paolo Tonin, Giacinto Bagetta, Maria Tiziana Corasaniti

**Affiliations:** 1 Pharmacotechnology Documentation and Transfer Unit, Preclinical and Translational Pharmacology, Department of Pharmacy, Health and Nutritional Sciences, University of Calabria, 87036 Rende, Italy; 2 Regional Center for Serious Brain Injuries, S. Anna Institute, 88900 Crotone, Italy; patonin18@gmail.com; 3 Center for Clinical Pharmacology and Pharmaceutics, Nihon Pharmaceutical University, Saitama 362-0806, Japan; k-hamamura@daiichi-cps.ac.jp; 4 Department of Physiology and Anatomy, Faculty of Pharmaceutical Sciences, Tohoku Medical and Pharmaceutical University, Sendai 981-8558, Japan; w-chizu@tohoku-mpu.ac.jp; 5 Department of Health Sciences, University “Magna Graecia” of Catanzaro, 88100 Catanzaro, Italy; mtcorasa@unicz.it

**Keywords:** transgenic mice, DYT1, dystonia, torsinA, pain, gabapentin, neurodegenerative diseases

## Abstract

Murine models are fundamental in the study of clinical conditions and the development of new drugs and treatments. Transgenic technology has started to offer advantages in oncology, encompassing all research fields related to the study of painful syndromes. Knockout mice or mice overexpressing genes encoding for proteins linked to pain development and maintenance can be produced and pain models can be applied to transgenic mice to model the most disabling neurological conditions. Due to the association of movement disorders with sensitivity and pain processing, our group focused for the first time on the role of the torsinA gene GAG deletion—responsible for DYT1 dystonia—in baseline sensitivity and neuropathic responses. The aim of the present report are to review the complex network that exists between the chaperonine-like protein torsinA and the baseline sensitivity pattern—which are fundamental in neuropathic pain—and to point at its possible role in neurodegenerative diseases.

## 1. Dystonia and Pain

It is important to investigate pain since it represents one of the most frequent causes of need for clinical assistance [1]. In particular, neuropathic pain is very debilitating, and neuropathies caused by comorbidities, such as diabetes, are very common, with a prevalence of 60% [2]. DYT1 dystonia is a severe early-onset inherited neurologic hyperkinetic movement disorder affecting the basal ganglia and cerebellum [3,4]; torsinA is expressed in the γ-amino butyric acid (GABA)-ergic and glutamatergic neurons of the dorsal horn superficial *laminae* [5], in which torsinA colocalizes with the α2δ-1 L-type voltage-dependent calcium channel subunit that is upregulated during the course of neuropathic pain (MT Corasaniti, personal communication). Therefore, a link exists between genetic movement disorders, thermal hyperalgesia and pain. The G_o_ knockout (KO) murine model is characterized by hyperalgesia on the hot-plate test accompanied by severe motor control impairment and hyperactivity, which is observed as counterclockwise turning behavior [6]. Moreover, disabling pain occurs in 56–62% cases of dystonia [7] in the affected region, reducing quality of life [8,9]; 70% of patients experiences cervical dystonia (*torticollis*); 30% blepharospasm; and 32% focal hand dystonia and writer’s cramp [8]. Pain–pressure thresholds are halved in idiopathic cervical dystonia, with a diurnal increase in pain intensity that reaches a *plateau* in the evening [10]. Furthermore, alterations in descending modulation [11] and axonal sensory polyneuropathies [12,13] can occur. Botulinum toxin (BoNT)—used in the preventative treatment of chronic migraines as a novel monoclonal antibody directed towards the calcitonin gene-related peptide (CGRP) or its receptor [14]—can offer relief [15] from dystonia-related pain. This provides proof that the latter painful syndromes involve the descending pathway, which modulates the perception of pain stimuli through serotonergic and noradrenergic neurotransmission; furthermore, these syndromes are not characterized by musculoskeletal pain only [11]. Another interesting common feature between pain and dystonia is the finding that 25% of patients suffering from complex regional pain syndrome type 1 (CRPS-1) develop permanent disabling dystonia [16,17]. CRPS-1 is a painful syndrome that can occur after stroke when a nerve lesion is not identifiable; type II CRPS occurs when a definite nerve lesion is identified. Incidentally, when adult-onset focal dystonia is associated with pain, the co-occurrence of severe neuropsychiatric symptoms that require accurate assessment to offer the adequate management and improvement of quality of life has been described [18], for example, in Parkinson’s and Alzheimer’s disease (PD and AD) [19,20,21]. PD and AD are neurodegenerative diseases characterized by alterations in movement and cognitive deterioration, respectively. 

## 2. TorsinA

TorsinA is a ubiquitous chaperonine-like protein of the endoplasmic reticulum that has been implicated in cytoskeletal dynamics [22] and stress-induced endoplasmic reticulum-associated degradation [23]; in addition, it is also involved in the release of neurotransmitters [24,25], being localized in the cerebellar cortex, nuclei and striatum [25]. In, fact, torsinA undergoes post-transcriptional upregulation following acute endoplasmic reticulum stress. Although efforts to elucidate the role of torsinA have focused mainly on its involvement in protein unfolding or disassembly machinery due to its homology to bacterial proteins with this function, its critical role at the nuclear envelope has now been recognized. In particular, torsinA is a luminal protein that resides within the contiguous lumens of the endoplasmic reticulum and nuclear envelope. Despite what was originally assumed [26], torsinA molecules cannot exert their biological activity alone; they require two ATPase activating cofactors with luminal domains [27], including lamina-associated polypeptide 1 (LAP 1) in the inner nuclear membrane [28] and luminal domain-like LAP1 (LULL1) in the endoplasmic reticulum projecting into the cytoplasm [29]. Therefore, these proteins, also known as TOR1AIP1 and TOR1AIP2, respectively, are needed to activate torsinA [30]; they can control the activities of several members of the Torsin superfamily, which reside in the endoplasmic reticulum and the perinuclear space [31]. This localization explains the involvement of torsinA in the fusion of the inner and outer nuclear membranes during interphase nuclear pore complex biogenesis, as well as in nuclear–cytoplasmic transport and in the linkers of nucleoskeleton and cytoskeleton (LINC) complex-dependent nuclear-cytoskeletal coupling [32,33,34,35]. Therefore, dystonia may be characterized by a loss of function of this control mechanism [27].

DYT1 transgenic rats present with an increase in the phosphorylation of eukaryotic initiation factor 2 (eIF2α), which is required for translation initiation and involved in an abnormal response of the endoplasmic reticulum to acute stress [36]; the dysregulation of eIF2α has been confirmed by transcriptomic analysis [36]. Premature long-term potentiation (LTP), which alters the formation of memory, and an increase in the levels of pro-brain-derived neurotrophic factor (BDNF) and BDNF have been found in these murine models of dystonia [37]. Moreover, long-term depression (LTD) in these transgenic mice is lost in contrast to strengthened LTP in striatal spiny projection neurons that present an increased function of the α-amino-3-hydroxy-5-methyl-4-isoxazole-propionic acid (AMPA) receptor, a reduction in the N-methyl-D-aspartate (NMDA)/AMPA ratio and increased levels of proteins associated with endoplasmic reticulum stress [38]. In fact, in the striatum, it is possible to notice a disinhibition of GABA-ergic synaptic activity [39]; an accumulation of AMPA receptors in spiny neurons [37]; an increase in the levels of mu opioid receptors [40]; and the inhibition of cholinergic interneuron transmission [40]. This is corroborated by the finding that the µ opioid receptor agonist tapentadol reduces heat hyperalgesia in diabetic neuropathic mice [41,42] and that perampanel, which acts as an antagonist of the AMPA receptor, attenuates the heat hyperalgesia caused by a neuropathic pain model of chronic constriction injury [43]. Along with these modifications, aberrant cerebellar changes are found in the developmental period [44]. In agreement with the alterations in movement, there is an impairment in D2 dopaminergic signaling [45,46,47,48,49] indicating an imbalance between dopaminergic and cholinergic tone [50], which can be counteracted by adenosine A2A receptor antagonism [51] together with modifications to the release or transport of dopamine [52], as demonstrated via pharmacological tool amphetamine. However, the role of torsinA in dopamine release has not yet been elucidated [53]. 

## 3. Different Transgenic Models of Dystonia

**Figure 1:**
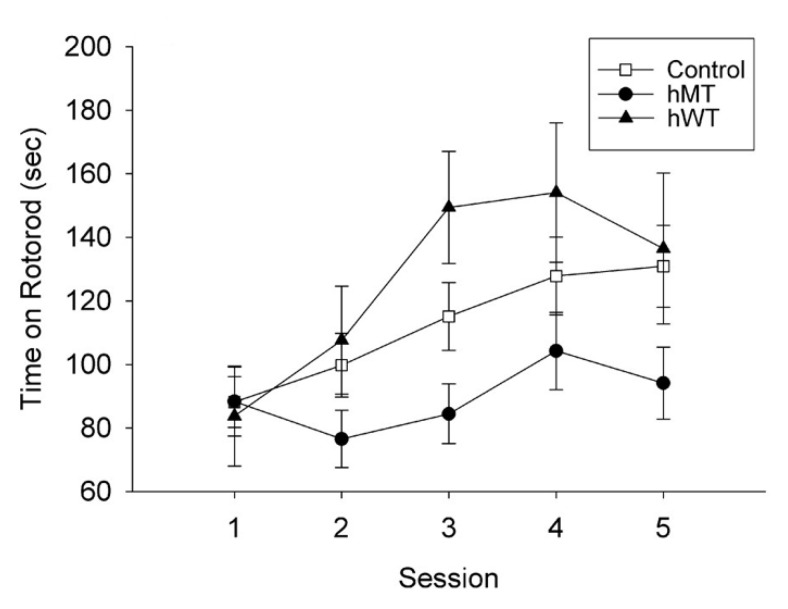
Effect of the overexpression of human wild-type (hWT) and mutated (hMT) torsinA on locomotor activity with respect to non-transgenic littermates (control). The transgene-positive hMT mice fell off the rotarod sooner than the hWT mice (*p* < 0.05). Reproduced with permission from [57].

Various transgenic models offer the opportunity to identify common features and targets for pharmacological intervention in the distinct forms of dystonia [54]. The tor1A gene ΔGAG deletion causes the loss of a residue of glutamic acid in the carboxy-terminal region of torsinA [55], a protein included in the AAA+ATPase family that is associated with a variety of cellular activities [56] implicated in the neurodevelopmental disease DYT1 dystonia. Transgenic murine models of DYT1 dystonia can be produced by expressing the gene encoding the human mutant (hMT) or the human wild-type (hWT) torsinA [57]. In particular, hWT or hMT torsinA cDNA is inserted into a pcDNA3.1 vector under the human cytomegalovirus early promoter, linearized and introduced into fertilized B6C3Fq eggs via microinjection to obtain founder mice to backcross with the C57BL/6 strain [57,58]. At 9 months, the hMT mice present a decrease in learning and reduction in motor activity [57] (Figure 1).

DYT1 ΔGAG knock-in (KI) mice show reduced levels and binding activities of the D1 and D2 receptors [59]; on the other hand, dopamine receptor 1-expressing cell-specific DYT1 conditional KO mice display the defective maturation of D1 receptors, a reduction in spontaneous locomotor activity, alterations in gait and a decrease in slips during the beam-walking test [59]. Adenosine A2A receptor expression in transgenic mouse models of DYT1 dystonia is increased in the striatum and the globus pallidus, in particular in cholinergic interneurons, and reduced in the entopeduncular nucleus [60]. Furthermore, DYT1 transgenic mice present sparse and small D2 synapses in the striatum that are hypothesized to be insufficient to manage presynaptic dopamine release [49]. The ΔGAG KI mice present reduced levels of α-synuclein in glutamatergic striatal terminals together with an imbalance in synaptic N-ethylmaleimide sensitive fusion attachment protein receptor proteins (SNARE, responsible for Ca^2+^-dependent exocytosis), which causes vesicle recycling alterations due to their role in the docking and priming stages of exocytosis [61] and thus has an impact on synaptic communication and aberrant plasticity [62]. In fact, SNARE proteins, together with synaptotagmin I, require proper targeting into specific regions of the presynaptic neuron that define the sites for fusion initiation to exert their function [63]; in addition, some are involved in the regulation of glutamate receptors [64]. In particular, synaptosomal-associated protein (SNAP)-25 is clustered in specific regions that overlap in part with syntaxin, which is clustered in cholesterol-dependent sites at which secretory vesicles dock and fuse. Furthermore, SNAP-23 is expressed in soma and dendrites, and studies using transgenic animals point at its role in the surface expression and membrane recycling of NMDA receptors. Moreover, it has been shown that torsinA protein levels—assessed via Western blot analysis—are reduced by 47% in the dorsal striatum of α-synuclein-null mice, suggesting a reciprocal modulatory interaction [62]. Patch-clamp recording experiments carried out in the DYT1 murine model measuring spontaneous inhibitory (GABA-mediated) and excitatory (glutamate-mediated) postsynaptic currents have recorded decreased asynchronous release from striatal spiny neurons [62]. D2 receptor-expressing cell-specific DYT1 conditional KO mice, used as a transgenic model of DYT1 early-onset generalized torsion dystonia, highlight locomotor deficits that are demonstrated in the accelerated rotarod and beam-walking tests and a significant reduction in striatal torsinA, acetylcholine metabolic enzymes, tropomyosin receptor kinase A (trk A), cholinergic interneurons, dimers of D2 receptors and tyrosine hydroxylase [65]. Selective transgenic models of DYT1 obtained via the knockout of D2 receptor-expressing neurons (D2KO) or only cholinergic neurons (ch2KO) show that the loss of function mutation in torsinA (which results in reduced sensory-evoked brain activity within the sensory motor network and the impairment of the functional connectivity of the striatum that is correlated with motor alterations) is associated with worse defects if it occurs in medium spiny and dopaminergic neurons of the basal ganglia as opposed to cholinergic neurons [66].

In contrast to transgenic mouse models of DYT1 dystonia, the GNAL rat model of DYT25 dystonia does not present alterations in the striatal levels of α-synuclein [62]. The GNAL gene encodes for the guanine nucleotide-binding protein G (olf) subunit alpha (Gαolf), which is a modulator in the olfactory bulb and the striatum and functions in neurotransmission involving D1 and adenosine A2A receptors. This transgenic model expresses a reduction in locomotor activity, impaired rotarod performance and abnormal motor learning, which are related to AMPA receptors and the downregulation of activity-regulated cytoskeleton-associated protein (arc) [67]. LTD is lost in striatal spiny projection neurons obtained from transgenic mouse models of DYT1 dystonia as well as from the GNAL rat model of DYT25 dystonia [54]. A transgenic mouse in which the expression of mutant torsinA in the forebrain was restricted to striatal medium spiny neurons and cerebellar Purkinje cells under the control of the darpp-32 gene fragment D9 [68] showed alterations similar to the pancellular DYT1 transgenic mice [69], including a non-cell autonomous effect on dopamine release from striatonigral axons and the expression of mutant nigral dopaminergic neurons and cholinergic interneurons, despite not showing differences in open field, rotarod, staircase reach and beam walking tests when compared to the non-transgenic littermates [69]. In addition, cholinergic interneuron-specific DYT1 conditional KO mice (DYT1 ch2KO; from which the neomycin cassette is removed with respect to the original model to avoid ectopic recombination) show the following defects [70]: the impairment of paw clenching behavior; deficits in motor coordination and impaired motor learning; and a decrease in the number of striatal cholinergic interneurons, with altered current density and pharmacological modulation, and of the levels of striatal choline acetyltransferase. Dystonia transgenic models have allowed for the identification of potential therapeutic targets, e.g., the M4 muscarinic receptors located on striatal cholinergic interneurons [71] and the phosphatidic acid phosphatase lipin [72]. A dlx conditional KO model of DYT1 dystonia at postnatal day 14 showed the accumulation of perinuclear ubiquitin in the reticular thalamic nucleus, ventral forebrain and cortex neurons; this accumulation also occurred in forebrain GABAergic neurons expressing the dlx5/6-cre transgene, which was also present in cholinergic neurons in the relevant model [73]. Furthermore, dysfunctional nuclear pore complexes, abnormal perinuclear ubiquitin accumulation, the alteration of the inner nuclear membrane and lamina proteins and nuclear pore complex clustering have been found, and some of these defects can persist into adult age [73]. Transgenic models of myoclonus dystonia (DYT11) exist and have undergone improvements to obtain the acute knockdown of the scge gene, allowing for the initiation of dystonia and repetitive, myoclonic-like, jerking movements that sensitive to ethanol, as opposed to the first models that displayed only mild behavioral phenotypic alterations [74]. The deletion of both torsinA and torsinB with emx1-cre, an endogenous emx1 locus that directs the expression of cre recombinase, in the emx1 (a + b) conditional KO transgenic model causes cell loss and reactive gliosis in the cre-expressing cerebral cortex and hippocampus; in addition, the cortex is significantly thinner and the deletion of torsinB increases the susceptibility to the loss of function of torsinA, thus worsening the motor and neuropathological phenotypes induced by the deletion of torsinA [75]. These findings highlight the possible role of torsinB in dystonia pathogenesis [75]. DYT6 dystonia is due to mutations in the transcription factor Thanatos-associated protein 1 (thap1), which is a ubiquitously expressed transcription factor with DNA-binding and protein-interaction domains; furthermore, thap1-null conditional KO mice present with dysfunctions in the dopaminergic indirect pathway [76]. Incidentally, a heterozygote thap1 c54y or Δexon2 allele in the striatum and cerebellum confers alterations similar to those typical of DYT1 dystonia, including [77] eIF2α signaling, glutamate-induced LTD, synaptic plasticity and neuritogenesis. KO mutant mice with mutations in the GTP cyclohydrolase 1 gene (gch1) used as models of DYT5 dystonia display abnormal movements and dystonic posture in the hindlimbs in parallel with tyrosine hydroxylase depletion in the striatum [78]. In addition, heterozygous mice carrying a point mutation in the atp1a3 gene, which encodes the catalytic subunit (α3) of the Na^+^/K^+^ ATPase pump, represent a transgenic model of rapid-onset dystonia parkinsonism (DYT12), showing spatial learning impairments and hyperactivity, while significant motor deficits in beam-walking and rotarod tests have been highlighted in females [79]. 

The main similarities and differences among the several dystonias are summarized in Table 1.

## 4. Transgenic Modelling Is Fundamental for the Translational Study of Pain: Neuropathic Pain in hMT and hWT Transgenic Mice

Murine models are necessary for the study of clinical conditions and for the development of drugs and treatments [81]. In fact, the first transgenic animal models and immunocompromised mice not bearing spontaneous mutations date back to the study of antiblastic chemotherapies for cancer treatment [82]. Preclinical mouse models using, in particular, transgenic and KO technology have allowed for advances in the development of therapies in cancer, making pharmacokinetic, pharmacodynamic and toxicology studies of novel therapeutic agents possible [82]. Although it is still intensely debated that animal models do not always recapitulate human disease, they permit faster progress in the investigation of novel therapies, which is impeded by clinical trials [82] because of the following issues: high rates of compound attrition; a small number of patients meeting the inclusion criteria in definite conditions; long enrollment periods; and long waiting periods during Phase I–III. In oncology, xenograft studies have shown some predictivity despite their deficiencies [83], which are mostly solved by genetically engineered mouse models [82]. Therefore, an accurate characterization of preclinical models is necessary to provide reliable preclinical studies investigating synthetic and natural compounds [84,85,86,87] and form a sound rational basis for clinical translation in the study of diseases relevant to human research. In this way, only the compounds with strong potential to obtain meaningful results are investigated in clinical trials, which require a long, complex, rigorous process to obtain high quality evidence [88,89,90,91,92].

The use of transgenic technology has also gained growing interest in research on pain, with the production of KO mice or mice overexpressing genes encoding for proteins linked to pain development and maintenance, including neurotrophins, which are responsible for the growth of neurons, and their receptors; peripheral mediators of nociception and hyperalgesia; and opioids and non-opioid neurotransmitters, as well as their receptors and intracellular signal transduction molecules [93]. It is fundamental to study novel pharmacological and pharmacotherapeutic agents; however, transgenic technology is also used in new approaches to circuit mapping for transneuronal labeling to trace pain and descending inhibitory circuits [94]. Mice overexpressing nerve growth factor (NGF) in the skin showed an ectopic network of sensory fibers in the spinal cord containing the excitatory neurotransmitter substance P [95], which was associated with increased mechanical sensitivity as assessed by Von Frey’s test [96] (an evaluation of baseline mechanical sensitivity and allodynia due to pain conditions that uses the application of calibrated filaments with incremental strength on the affected paw through the up-down method), profound hyperalgesia due to noxious mechanical stimulation [97] and thermal allodynia and hyperalgesia [95], in contrast to transgenic mice underexpressing NGF that were hypoalgesic [97]. The latter increased sensitivity was reversed by substance P and N-methyl-D-aspartate receptor (NMDA) antagonists [95]. Heat thermal sensitivity and hyperalgesia have been investigated in several transgenic mice models of disabling neurological diseases. Double transgenic mice overexpressing the amyloid precursor protein (app) and presenilin-1 (psen-1) genes (tastpm), which are a preclinical model of familial AD, displayed an increased latency to heat in parallel with cognitive deficits at 6 months [98]. Although it is not an induced mutation, aging can prompt alterations in pain processing [99,100,101], including an increased sensitivity [102,103] and response to analgesics for neuropathic pain treatment [99], as well as behavioral disturbances that are characteristic of dementia [104]. Moreover, thermal hyperalgesia has been described in the mecp2-308 murine model of Rett syndrome [105], a neurodevelopmental disease characterized by rigidity and spasticity; 90–95% of cases are due to mutations in the methyl-CpG-binding protein 2 (mecp2) gene [106]. KO mice for the tachykinin neuropeptide substance P and its pro-peptide precursor preprotachykinin-A (ppt-A) and derived peptides do not show differing results in pain tests without supraspinal involvement, such as the tail flick assay and acetic acid-induced writhing test, while they show analgesia in the formalin test [107] (the intraplantar administration of formalin, inducing a biphasic licking/biting nocifensive response due to local pain and central sensitization). Studies on transgenic mice with mutations affecting the opioid system deserve further investigation [108], even in light of the paucity of trials with specific populations, e.g., post-stroke pain patients [109], as does the role of the 5-HT1B autoreceptor in sensitivity to morphine, which has been demonstrated through inbred mice [110].

**Figure 2:**
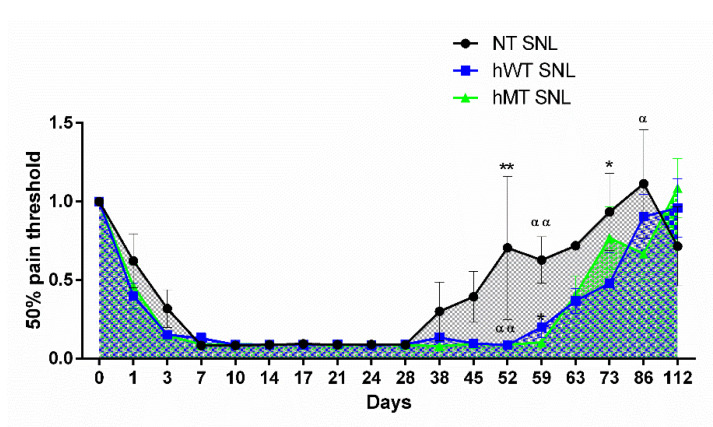
Time course of spinal nerve ligation (SNL)-induced mechanical allodynia in mice overexpressing human wild-type (hWT) and mutated (hMT) torsinA. Non-transgenic (NT), hWT and hMT mice did not show statistically significant differences in the development and maintenance of mechanical allodynia. However, hWT and hMT mice presented a delayed recovery from sensitization with longer lasting mechanical allodynia (two-way ANOVA F (32,374) = 1.561; *p* < 0.05 *; day 52 hWT vs. NT *p* < 0.01 **, hMT vs. NT *p* < 0.01 ^αα^; day 59 hWT vs. NT *p* < 0.05 *, hMT vs. NT *p* < 0.01 ^αα^; day 73 hWT vs. NT *p* < 0.05 *; day 86 hMT vs. NT *p* < 0.05 ^α^). Data are expressed as mean ± SEM of the nociceptive reaction. *p* values < 0.05 were considered statistically significant. *n*: NT = 4; hWT = 10; hMT = 11. Reproduced with permission from [5].

Due to the link between dystonia and the molecular machinery involved in pain processing, previous research studies from our group compared for the first time the patterns in the baseline sensitivity and neuropathic responses of hWT and hMT mice with that of non-transgenic (NT) mice. The results demonstrated that baseline mechanical sensitivity did not significantly differ among the three types of mice [5]. The induction of neuropathic pain through the ligation of the L5 spinal nerve (SNL) induced prolonged mechanical allodynia, as measured via Von Frey’s test, in the transgenic mice modelling dystonia (Figure 2) [5]. 

In conflict with mechanical sensitivity, baseline thermal sensitivity was increased in hMT mice, although no statistically significant differences in hyperalgesia induced by SNL were observed; only a trend towards decreased heat thresholds was found in the hWT and hMT mice [111]. Since GABA-ergic inhibitory control is needed to avoid aberrant processing within the dorsal horn after nerve injury [112], these results suggest that the transgenic murine model of dystonia exhibited delayed recovery from the sensitization process because of the role of torsinA in the activity of GABA-ergic and glutamatergic interneurons, the development of abnormalities in the neuronal nuclear membrane [113] and synaptogenesis [44]. Moreover, BDNF is of the utmost importance to the development of central sensitization during pain; it is released by primed microglia in *lamina* I neurons during neuropathic pain and allodynia [114], and its levels are modulated by torsinA [37]. Moreover, torsinA might be involved in opioid signaling [40], as suggested by its colocalization with α2δ-1. In fact, α2δ subunits can alter the release of Ca^2+^ from the endoplasmic reticulum [112], possibly interfering with the function of proteins responsible for calcium channel trafficking [115,116]. This corroborates our data demonstrating the enhanced effectiveness of gabapentin in hMT mice [5]. Gabapentin, pregabalin and the novel mirogabalin [117,118] are scarcely used in non-communicative patients [119,120,121], and the co-occurrence of different responses to these drugs with neuropsychiatric symptoms prompts the engineering of clinical trials to assess the efficacy and safety of analgesic essential oils for pain and behavioral disorders [122,123,124,125] in dystonic patients in order to reduce adverse reactions. The observed differences between the patterns of thermal sensitivity and hyperalgesia and mechanical sensitivity could be dependent on the diverse mechanisms of development of these phenomena. Thermal hyperalgesia to heat is supposed to be primary and not secondary—caused by alterations in central pain processing [126], because of the lack of facilitation of inputs from heat sensitive nociceptors—and involves transient receptor potential vanilloid 1 (TRPV1) channels [127]. To the contrary, mechanosensitive pathways involve central sensitization [128]. In particular, heat hyperalgesia has been suggested to be absent in the secondary zone with respect to the injury primary zone [128]. Since the DYT1 dystonia transgenic mouse model displays cholinergic dysfunction in the dorsal striatum [129], as in Parkinson’s and AD, the possible role of the human, wild-type and mutated genes encoding torsinA in sensitivity and chronic pain in neurodegenerative diseases deserves further investigation. In particular, the influence of the possible alteration of its role in functions related to the nuclear envelope on pain processing and maintenance needs to be deeply studied at the molecular and phenotypic level.

## Figures and Tables

**Table 1:** Summary of genes and proteins involved in the genetic forms of primary dystonia and dystonia syndromes. Adapted and reproduced with permission from [80].

Locus	Designation	Clinical Features	Gene/Inheritance	Protein	Putative Functions
Pure Dystonia					
DYT1 Chr9q34.11	Early-onset primary dystonia	Childhood onset dystonia in limb with generalization	tor1A autosomal dominant	torsinA	AAA+ protein, nuclear envelope, endoplasmic reticulum secretory and stress response, regulation of synaptic function
DYT2	Early-onset dystonia	Adolescent-onset segmental or generalized	Autosomal recessive	Unknown	
DYT4 Chr19p13.3	Whispering dysphonia	Childhood-onset laryngeal abductor spasm with cervical dystonia	tubb4a autosomal dominant	beta-tubulin 4a	Structural cytoskeleton protein
DYT6 Chr8p11.21	Autosomal dominant early-onset focal dystonia	Early-onset dystonia with prominent cervical and laryngeal involvement	thap1 autosomal dominant	Thanatos-associated domain-containing apoptosis associated protein 1	Atypical zinc-finger protein; thap domain is chromatin-binding factor and regulates transcription
DYT7 Chr8p	Familial focal dystonia	Adult-onset focal dystonia	Unknown autosomal dominant	Unknown	
DYT13 Chr1p36.32– p36.13	Familial craniocervical dystonia	Focal or segmental dystonia of the craniocervical region and upper limbs	Unknown autosomal dominant	Unknown	
DYT17 Chr20p11.2– 2q13.12	Early-onset autosomal recessive dystonia	Early-onset focal dystonia progressing to generalized with dysphonia and dysarthria	Unknown autosomal recessive	Unknown	
DYT21 Chr2q14.3– q21.3	Late-onset dystonia	Late-onset multifocal and generalized dystonia	Unknown autosomal dominant	Unknown	
DYT23 Chr9q34.11	Cervical dystonia	Late-onset primary cervical dystonia	ciz1 autosomal dominant	cip1-interacting zinc finger protein 1	Regulation of G1–S cell cycle and DNA replication
DYT24 Chr11p14.2	Late-onset dystonia	Cranial and cervical dystonia	ano3 autosomal dominant	Anoctamin 3	Calcium-gated chloride channel
DYT25 Chr18p	Cervical dystonia with local spread	Predominantly late-onset primary cervical dystonia with spread to face	gnal autosomal dominant	Alpha subunit of G protein	Probable interaction with D1 and adenosine 2A receptors.
**Dystonia syndromes**					
DYT3 Xq13.1	X-linked dystonia (Lubag)	Segmental or generalized dystonia with parkinsonism	taf1 X-linked	TATA box-binding protein associated factor 1	Regulation of transcription initiation and cell cycle
DYT5/14 Chr2q13.2	DOPA (precursor of dopamine)-responsive dystonia	Dystonia with parkinsonism, diurnal variation, and very good response to L-dopa	gch1 autosomal dominant	GTP cyclohydrolase 1	Rate-limiting enzyme in synthesis of tetrahydrobiopterin, key cofactor in monoamine synthesis; results in deficient dopamine synthesis
DYT11 Chr7q21.3	Myoclonic dystonia syndrome	Upper body myoclonic jerks with dystonia; responsive to alcohol	sgce autosomal dominant	Epsilon-sarcoglycan	Cell membrane protein that may act as structural platform for other protein interactions
DYT12 Chr19q13.2	Rapid-onset dystonia parkinsonism	Acute-onset generalized dystonia with parkinsonism; rostrocaudal gradient of symptoms	atp1a3aAutosomal dominant	Alpha 3 subunit of Na/K ATPase	Subunit of Na/K ATPase on neuronal membrane
